# Bridging Awareness and Acceptance of Pre-Exposure Prophylaxis Among Men Who Have Sex With Men and the Need for Targeting Chemsex and HIV Testing: Cross-Sectional Survey

**DOI:** 10.2196/13083

**Published:** 2019-07-03

**Authors:** Tsz Ho Kwan, Shui Shan Lee

**Affiliations:** 1 Jockey Club School of Public Health and Primary Care The Chinese University of Hong Kong Shatin China (Hong Kong); 2 Stanley Ho Centre for Emerging Infectious Diseases The Chinese University of Hong Kong Shatin China (Hong Kong)

**Keywords:** pre-exposure prophylaxis, men who have sex with men, HIV, health knowledge, attitudes, practice, psychotropic drugs

## Abstract

**Background:**

Pre-exposure prophylaxis (PrEP) is currently an important tool for HIV prevention, especially in communities with higher risk of infection, notably men who have sex with men (MSM). To date, PrEP has remained generally unavailable in many cities around the world. In the planning of strategies for PrEP targeting MSM, community assessment is crucial to understand members’ responses to the new intervention.

**Objective:**

Awareness and acceptance are 2 different but intricately linked contexts of PrEP. The aim of this study was to identify the determinants of awareness and acceptance of PrEP among MSM and to delineate their interrelationships in Hong Kong where PrEP services have not been developed.

**Methods:**

A Web-based questionnaire survey was administered in light of the popularity of the internet as a platform for information and networking in the MSM community. Factors associated with PrEP acceptance and awareness were separately analyzed, and their predictors were subsequently tested by multivariate logistic regression. Associations between acceptance and awareness of PrEP were examined by factor network analysis.

**Results:**

Between August and September 2016, results from a total of 453 HIV-negative MSM were analyzed. Half (49.7%, 225/453) of the respondents were aware of PrEP, and 78.3% (355/453) would consider using PrEP when it becomes available. Awareness of PrEP was associated with recent (*P*=.01) and ongoing (*P*=.04) use of psychotropic drugs for sex (chemsex). MSM who used online forums to seek sex partners had lower awareness (*P*=.04) than those visiting physical venues for sex networking. MSM who accepted PrEP were more likely users of internet channels for sex networking (*P*=.049), especially location-based social network apps (*P*=.04). MSM accepting PrEP were more concerned about their partners’ HIV status (*P*=.002), history of sexually transmitted infections (*P*=.01), condom use (*P*=.02), and HIV testing behavior (*P*=.02). Multivariate logistic regressions revealed that PrEP awareness was related to one’s networking pattern, whereas its acceptance was associated with inclination to self-protect from HIV. Factor network analysis highlighted the importance of chemsex, which was linked by over half of the edges, whereas the rest were contributed by HIV testing behaviors.

**Conclusions:**

In Hong Kong, the overall awareness among MSM toward PrEP was only moderate but their acceptance was higher. Targeting MSM with chemsex behaviors through Web-based platforms and parallel development of tailored HIV testing services are important when introducing PrEP in the community.

## Introduction

Globally, the HIV epidemic is continuing to grow among men who have sex with men (MSM). In Hong Kong, a notable increase in the number of MSM diagnosed with HIV infection has been seen since 2004 [1], contributing to 63% of all reported new cases in 2017 [2]. Although condom use is the most important measure to prevent HIV infection, its rates were suboptimal, ranging from 60% to 80% for the last anal sex with various types of partners [3]. Having condomless sex was associated with lower self-efficacy of consistent condom use, higher perceived barriers against condom use [4], and other situational and environmental phenomena, such as alcohol use, overseas sex, and not at home [5]. Despite its high efficacy for HIV prevention, barriers to condom use commonly exist. In circumstances where a condom is unavailable or not used for various reasons, other measures against HIV infection would need to be in place. To *end AIDS by 2030*, The Joint United Nations Programme on HIV/AIDS proposed that, inter alia, 90% of people by 2020 should have access to a package of prevention options, the latter referring to a multitude of measures, including pre-exposure prophylaxis (PrEP) [6].

PrEP was first approved for HIV prevention by the US Food and Drug Administration in 2012, followed by recommendations by the World Health Organization in 2014 [7]. Since then, demonstration projects grew rapidly worldwide, although the uptake has been slow [8]. Before rolling out PrEP in the community, it is important to assess MSM’s awareness and acceptance and their relationships with demographics and sexual and networking behaviors. In Asia and the Pacific, awareness of PrEP in the MSM community has so far been low (5% in Myanmar [9], 11% in China [10], 40% in Taiwan [11], and 44% in Malaysia [12]) and their acceptance of PrEP varied (39% in Malaysia [12], 56% in Taiwan [13], 62% in Myanmar [9], and 68% in China [10]). Awareness and acceptance are 2 different yet interrelated contexts of PrEP. Studies suggested that HIV testing history, demographic characteristics, networking pattern, and perceived discrimination against people living with HIV were associated with the magnitude of PrEP awareness [14,15]. Acceptance of PrEP was, on the contrary, associated with risk behaviors such as a higher number of sex partners and inconsistent condom use, use of psychotropic drugs for sex (chemsex), and perceptions about one’s own HIV risk and efficacy of PrEP [9-12,16]. Moreover, the association between awareness and acceptance of PrEP was inconsistently reported. Significant association was reported among MSM in Malaysia [12] and Taiwan [11], and a marginal effect was observed in China [10] but no relationship was found in the United Kingdom [14].

With the increasing availability of PrEP worldwide, it is anticipated that the awareness of PrEP among MSM would increase. Identifying the factors associated with not only awareness but also acceptance of PrEP is important so that maximum health benefits could be achieved by addressing the community’s specific health needs and sexual behaviors. This study aimed to assess the MSM community’s awareness and acceptance of PrEP and the factors associated with their interrelationships in Hong Kong, an Asia Pacific city where PrEP is not yet generally available. The study involved post hoc analyses of data collected from a cross-sectional study on the HIV testing behaviors of MSM. Results from this study will help inform promotion and intervention strategies and enhance the understanding of the health needs of potential PrEP users.

## Methods

### Participants

The study was based on the post hoc analyses of data collected for an HIV prevention study targeting MSM living in Hong Kong. The details of survey administration and primary analysis have been described previously [17]. Briefly, between August and September 2016, MSM were recruited through an online forum and location-based social network (LBSN) mobile apps, the target audience of which were MSM. Subjects were eligible if they were male, had ever had sex with another male, were aged 18 years or above, were normally residing in Hong Kong, and were able to understand Chinese. An incentive in the form of an HK $25 coffee voucher (US $1=HK $7.8) was given upon completion of the questionnaire. Informed consent was obtained from all participants included in the study. This study was approved by the Survey and Behavioural Research Ethics Committee of The Chinese University of Hong Kong.

### Measures

#### Sociodemographic and Risk Profiles

The sociodemographic items collected were age, height, weight, ethnicity, place of origin, residing and working district, education level, employment status, monthly income, self-perceived body image types, sexual orientation and role, age of sexual debut, chemsex history and current habit, sexually transmitted infection (STI) history (including syphilis, gonorrhea, chlamydia, genital warts, herpes, and pubic lice), HIV testing history and recency, HIV status, and use of PrEP and post-exposure prophylaxis.

#### Sexual Behaviors With the Most Recently Acquainted Male Sex Partner

The time, location, and channel of getting to know the most recently acquainted male sex partner were gathered, with the partner’s characteristics as listed in the above subsection. With regard to the sex act, the corresponding location, venue, and condom and drug use were enquired.

#### Networking Behaviors

The use of internet channels or physical venues in the preceding 3 months was enquired. Internet channels included online forums and websites and LBSN targeting MSM and social groups in mobile communication apps such as Line, WhatsApp, and Telegram. Types of physical venues were sauna, bar, beach, public toilet, gym, massage center, and social functions.

#### Factors Considered When Choosing Male Sex Partners

A number of factors were asked if they had been taken into account when choosing male sex partners. If a factor was considered important, inquiry was made on the corresponding expected criteria, including age, height, weight, sexual role, body image type, sexual orientation, place of origin, ethnicity, education level, employment status, marital status, chemsex habit, HIV testing habit, HIV status, STI history, condom usage, and place of having sex.

#### Perceptions About Pre-Exposure Prophylaxis

Following a brief description of PrEP (Multimedia Appendix 1), awareness and acceptance of PrEP were assessed as the 2 key outcome measures in this study. Awareness was assessed by a trichotomized question, which could be translated as “before answering this questionnaire, how much did you know about PrEP?” One of the following 3 options could be chosen: *never heard of*, *was aware of it but did not know much*, and *had keen knowledge of*. The latter 2 responses were regarded as indicative of awareness of PrEP, whereas the first option was treated as unawareness. Acceptance was assessed by responses to questions on the preferred modality of PrEP (daily, event driven, time driven, or injection). Answers other than *would not consider taking PrEP* were regarded as willingness to use PrEP.

### Statistical Analyses

Self-reported HIV-positive participants were excluded from the analysis. All the variables included in the analyses were dichotomized, and their relationships with awareness and acceptance of PrEP were separately assessed with the chi-square test. If the expected count of a cell was less than 5, the Fisher exact test was used. A *P* value of less than .05 was considered significant in all analyses. To predict MSM’s awareness and acceptance of PrEP, significant factors were entered to separate multivariate logistic regression models and selected by backward elimination based on the likelihood ratio until the level of change became lower than .10. If a direct relationship between awareness and acceptance was not observed, their indirect relationships were explored by first identifying factors directly correlated with awareness and acceptance by significant phi coefficients. If no specific factors were associated with both awareness and acceptance, pairwise correlation between factors significantly associated with either awareness or acceptance were tested and only significant ones were retained, thus creating a bipartite graph, with 1 set of nodes representing variables significantly associated with awareness and another set with acceptance. Links (edges) connecting nodes between the 2 sets denoted significant associations. The variables’ network characteristics were measured by normalized degree centrality, that is, the proportion of edges connected to a particular node.

## Results

Of the 459 complete responses collected (completion rate=71%, of 647 nonduplicate responses collected), 6 self-reporting HIV-positive MSM were excluded. Data from the remaining 453 participants were available for analysis. Participants’ characteristics are shown in Table 1. Of them, 249 (55.0%) participants were aged 25 years or younger; 270 (59.6%) had a monthly income of less than HK $15,000; 249 (55.0%) and 131 (28.9%) were full-time workers and students, respectively; 79 (17.4%) attained secondary-level education or below; and 359 (79.2%) were gay. About two-thirds (293/453, 64.6%) of them had previously been tested for HIV. In total, 56 (12.4%) and 51 (11.3%) participants had been diagnosed with STIs and had chemsex, respectively. Three-quarters (346/453, 76.4%) of them had sought sex partners in the previous 3 months.

Overall, 225 (49.7%, N=453) MSM had ever heard of PrEP. MSM’s awareness of PrEP was not associated with their demographic characteristics (Table 2). History of HIV testing, STI diagnosis, and chemsex history were not associated with awareness either. Recent (odds ratio [OR] 2.64, 95% CI 1.01-6.94; *P*=.04) and current (OR 2.91, 95% CI 1.20-7.07; *P*=.01) engagement in chemsex were both associated with higher odds of PrEP awareness. Associations between MSM’s awareness of PrEP and their networking behaviors varied. Higher awareness was observed in MSM who, in the preceding 3 months, had sought partners outside Hong Kong (OR 1.55, 95% CI 1.02-2.34; *P*=.04), and it was lower in those who used online forums (OR 0.68, 95% CI 0.46-0.99; *P*=.04) for sex networking. An insignificant difference in the low awareness of MSM who sought partners in local physical venues was noted (33% vs 28%). MSM who were aware of PrEP were more likely to expect their partners to have a regular HIV testing habit (OR 1.90, 95% CI 1.23-2.92; *P*=.003). Partners’ health status, including HIV testing habit and status, and STI history were not associated with their awareness. Among these factors, recent chemsex habit (adjusted odds ratio [aOR] 3.28, 95% CI 1.32-8.14; *P*=.01), partner seeking through online forums (aOR 0.63, 95% CI 0.43-0.94; *P*=.02) and outside Hong Kong (aOR 1.66, 95% CI 1.08-2.54; *P*=.02) in the preceding 3 months, and expecting partners to be tested regularly (aOR 1.97, 95% CI 1.26-3.06; *P*=.003) were significant predictors of MSM’s awareness of PrEP.

Over three-quarters (78.4%, 355/453) of the participants responded that they would consider taking PrEP in the future. Acceptance was associated with working or studying fulltime (OR 2.19, 95% CI 1.27-3.78; *P*=.004) but not with other demographic variables (Table 2). MSM who had tested for HIV before (OR 1.68, 95% CI 1.06-2.65; *P*=.03) and those who had engaged in group sex (OR 1.68, 95% CI 1.04-2.69; *P*=.03) were more likely to accept PrEP. Those whose only partners in the past year were emotionally attached ones had a lower acceptance (OR 0.32, 95% CI 0.16-0.62; *P*<.001). MSM using internet channels (OR 1.61, 95% CI 1.00-2.60; *P*=.049), especially mobile apps (OR 1.60, 95% CI 1.01-2.51; *P*=.04), had a higher acceptance of PrEP. MSM who accepted PrEP considered their partner’s health status, including HIV testing habit (OR 1.99, 95% CI 1.13-3.52; *P*=.02), HIV status (OR 3.03, 95% CI 1.49-6.17; *P*=.002), STI history (OR 2.13, 95% CI 1.16-3.91; *P*=.01), and condom use habit (OR 3.88, 95% CI 2.10-7.17; *P*<.001), important when seeking partners. They also considered their partner’s education level (OR 1.66, 95% CI 1.05-2.62; *P*=.03) important. Though they were perceived to be important, expectations of the partner’s regular testing habit and consistent condom use were not associated with PrEP acceptance. In the multivariate regression model, working or studying fulltime (aOR 2.36, 95% CI 1.33-4.22; *P*=.004), having been tested for HIV (aOR 1.58, 95% CI 0.98-2.56; *P*=.06), having an emotionally attached partner as the only sex partner in the previous year (aOR 0.27, 95% CI 0.13-0.56; *P*<.001), and considering partner’s condom use habit important (aOR 4.08, 95% CI 2.15-7.75; *P*<.001) were predictors of PrEP acceptance.

**Table 1:** Characteristics of men who have sex with men in a cross-sectional survey in Hong Kong (N=453).

Variable		n (%)
**Age (years)**		
	≤25	249 (55.0)
	>25	204 (45.0)
**Monthly income**		
	<HK $15,000 (approximately US $1910)	270 (59.6)
	≥HK $15,000	183 (40.4)
**Employment status**		
	Full-time working	249 (55.0)
	Full-time studying	131 (28.9)
	Working or studying part-time, freelance, self-employed, unemployed, or retired	73 (16.1)
**Education level**		
	Secondary level or below	79 (17.4)
	Postsecondary level or above	374 (82.6)
**Sexual orientation**		
	Gay	359 (79.2)
	Bisexual	90 (19.9)
	Heterosexual	4 (0.9)
**HIV testing history**		
	Ever tested	293 (64.6)
	Never tested	160 (35.4)
**STI^a^ history**		
	Ever diagnosed with STI	56 (12.4)
	Never diagnosed with STI	397 (87.6)
**Chemsex history**		
	Ever had chemsex	51 (11.3)
	Never had chemsex	402 (88.7)
**Sought partners in the last 3 months**		
	Yes	346 (76.4)
	No	107 (23.6)

^a^STI: sexually transmitted infection.

**Table 2:** Univariate analysis of the factors associated with the awareness and acceptance of pre-exposure prophylaxis (N=453).

Variable		Aware of PrEP^a^				Will consider using PrEP			
		Yes (n=225)	No (n=228)	OR^b^ (95% CI)	*P* value	Yes (n=355)	No (n=98)	OR (95% CI)	*P* value
**Demographics**									
	Aged 25 years or below	130 (57.8)	119 (52.2)	1.25 (0.87-1.82)	.23	199 (56.1)	50 (51.0)	1.23 (0.78-1.92)	.38
	Monthly income <HK $15,000 (US $1=HK $7.8)	136 (60.4)	134 (58.8)	1.07 (0.74-1.56)	.72	208 (58.6)	62 (63.3)	0.82 (0.52-1.30)	.40
	Full-time working or studying	184 (81.8)	196 (86.0)	0.73 (0.44-1.21)	.23	307 (86.5)	73 (74.5)	2.19 (1.27-3.78)	.004
	Attained postsecondary or above education level	189 (84.0)	185 (81.1)	1.22 (0.75-1.99)	.42	299 (84.2)	75 (76.5)	1.64 (0.95-2.83)	.08
	Gay	183 (81.3)	176 (77.2)	1.29 (0.82-2.03)	.28	279 (78.6)	80 (81.6)	0.83 (0.47-1.46)	.51
**Risk profiles**									
	Ever tested for HIV	154 (68.4)	139 (61.0)	0.39 (0.94-2.05)	.10	239 (67.3)	54 (55.1)	1.68 (1.06-2.65)	.03
	Ever diagnosed with STI^c^	31 (13.8)	25 (11.0)	1.30 (0.74-2.28)	.36	47 (13.2)	9 (9.2)	1.51 (0.71-3.20)	.28
	Ever had group sex	98 (43.6)	88 (38.6)	0.85 (0.59-1.24)	.40	155 (43.7)	31 (31.6)	1.68 (1.04-2.69)	.03
	Ever had chemsex	30 (13.3)	21 (9.2)	1.52 (0.84-2.74)	.17	43 (12.1)	8 (8.2)	1.55 (0.70-3.42)	.27
	Having a chemsex habit currently	15 (6.7)	6 (2.6)	2.64 (1.01-6.94)	.04	18 (5.1)	3 (3.1)	1.69 (0.49-5.86)	.59^d^
	Last sex partner used drugs	19 (8.4)	7 (3.1)	2.91 (1.20-7.07)	.01	22 (6.2)	4 (4.1)	1.55 (0.52-4.62)	.43
	Boyfriend as the only sex partner in the previous year	19 (8.4)	20 (8.8)	0.96 (0.50-1.85)	.90	22 (6.2)	17 (17.3)	0.32 (0.16-0.62)	<.001
**Networking behaviors in the past 3 months**									
	Ever sought partners	177 (78.7)	169 (74.1)	1.29 (0.83-1.99)	.26	278 (78.3)	68 (69.4)	1.59 (0.97-2.62)	.07
	Sought partners outside Hong Kong	73 (32.4)	54 (23.7)	1.55 (1.02-2.34)	.04	107 (30.1)	20 (20.4)	1.68 (0.98-2.89)	.06
	Sought partners in local physical venues	74 (32.9)	63 (27.6)	1.28 (0.86-1.92)	.22	109 (30.7)	28 (28.6)	1.11 (0.68-1.81)	.68
	Sought partners through internet channels	166 (73.8)	161 (70.6)	1.17 (0.78-1.78)	.45	264 (74.4)	63 (64.3)	1.61 (1.00-2.60)	.049
	Sought partners in online forums	75 (33.3)	97 (42.5)	0.68 (0.46-0.99)	.04	133 (37.5)	39 (39.8)	0.91 (0.57-1.43)	.67
	Sought partners in mobile apps	153 (68.0)	136 (59.6)	1.44 (0.98-2.11)	.06	235 (66.2)	54 (55.1)	1.60 (1.01-2.51)	.04
**Factors considered important when choosing partners**									
	Partner’s HIV testing habit	197 (87.6)	189 (82.9)	1.45 (0.86-2.45)	.16	310 (87.3)	76 (77.6)	1.99 (1.13-3.52)	.02
	Expectation of partner’s regular HIV testing habit	181 (80.4)	156 (68.4)	1.90 (1.23-2.92)	.003	271 (76.3)	66 (67.3)	1.05 (0.50-2.22)	.89
	Partner’s HIV status	208 (92.4)	210 (92.1)	1.05 (0.53-2.09)	.89	335 (94.4)	83 (84.7)	3.03 (1.49-6.17)	.002
	Partner’s STI history	199 (88.4)	199 (87.3)	1.12 (0.63-1.96)	.71	319 (89.9)	79 (80.6)	2.13 (1.16-3.91)	.01
	Partner’s condom use habit	205 (91.1)	199 (87.3)	1.49 (0.82-2.73)	.19	329 (92.7)	75 (76.5)	3.88 (2.10-7.17)	<.001
	Expectation of partner’s consistent condom use habit	159 (70.7)	169 (74.1)	0.84 (0.56-1.27)	.41	266 (74.9)	62 (63.3)	1.74 (1.08-2.79)	.02
	Partner’s education level	118 (52.4)	102 (44.7)	1.36 (0.94-1.97)	.10	182 (51.3)	38 (38.8)	1.66 (1.05-2.62)	.03

^a^PrEP: pre-exposure prophylaxis.

^b^OR: odds ratio.

^c^STI: sexually transmitted infection.

^d^Fisher exact test.

**Figure 1:**
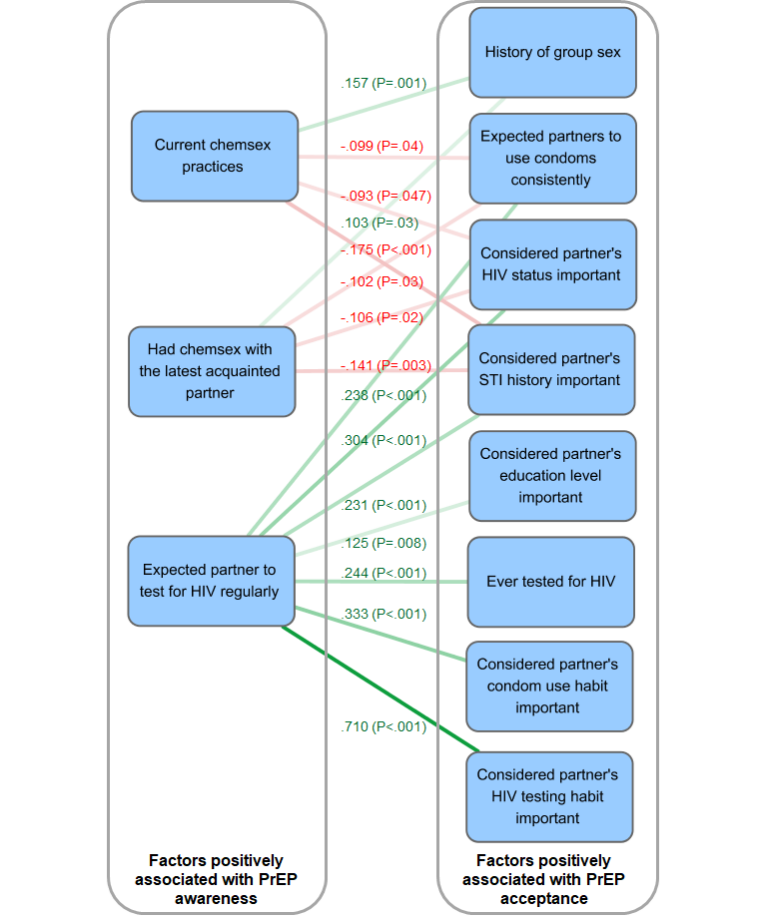
Bipartite factor network of variables associated with awareness and acceptance of pre-exposure prophylaxis among men who have sex with men in Hong Kong. Edge colors correspond to the labeled phi coefficients, where positive correlations are in green and negative ones are in red. STI: sexually transmitted infection; PrEP: pre-exposure prophylaxis.

Overall, 81% MSM who had heard of PrEP responded that they would consider taking PrEP. Among those who did not know about PrEP before, 76% indicated that they would consider it in the future after the brief introduction of PrEP. The association between PrEP awareness and acceptance was not significant (OR 1.35, 95% CI 0.86-2.11; P=.20). As awareness of PrEP was not directly associated with its acceptance, nor was there a variable significantly associated with both variables, correlations between factors separately associated with awareness and acceptance were tested (Figure 1). There were 3 and 8 factors that could be correlated with PrEP awareness and acceptance, respectively, constituting a total of 15 edges. Awareness was positively correlated with current (phi=0.096; *P*=.04) and recent (phi=0.116; *P*=.01) chemsex practices and expecting their partner to test for HIV regularly (phi=0.138; *P*=.003). The 2 chemsex-related variables had a normalized degree centrality of .267. They were both positively correlated with group sex history (phi=0.157; *P*=.001 and phi=0.103; *P*=.03) and negatively correlated with the expectation of sex partners to use condoms consistently (phi=−0.099; *P*=.04 and phi=−.102; *P*=.03) and with concerns on sex partners’ HIV status (phi=−0.093; *P*=.047 and phi=−0.106; *P*=.02) and STI history (phi=−0.175; *P*<.001 and phi=−0.141; *P*=.003). Conversely, the latter 3 variables were positively correlated with the expectation of partner’s regular testing habit (phi=0.238, *P*<.001; phi=0.304, *P*<.001; and phi=0.231, *P*<.001), which had the highest normalized degree centrality (.467). It was also positively correlated with considerations of partners’ education level (phi=0.125; *P*=.008), condom use habits (phi=0.333; *P*<.001) and HIV test habits (phi=0.710; *P*<.001), and one’s history of HIV testing (phi=0.244; *P*<.001).

## Discussion

### Principal Findings

In Hong Kong, where PrEP has not yet been formally introduced, only half of the MSM in the community were aware of PrEP, but a high proportion (78%) were willing to take PrEP should it become available. The moderate level of awareness observed was similar to that reported in Taiwan and Malaysia, where awareness levels were 40% and 44%, respectively [11,12]. However, the high acceptance was comparable with Mainland China [10], Brazil [18], and Kenya [19]. Although awareness and acceptance are 2 important attributes for engaging MSM in PrEP for achieving HIV prevention, their predictors were different. Our results suggest that awareness of PrEP is associated with one’s networking behaviors and risk profiles. Those who were aware of PrEP were more likely to be active on the Web and to seek partners overseas.

In the past few years, popular mobile LBSN apps in Hong Kong, such as Hornet, have incorporated a feature of stating the HIV status on each user’s profile [20]. Statuses including HIV negative, HIV positive, undetectable virus level, and on PrEP could be chosen. Early adopters who were using PrEP had most likely networked with overseas MSM at a time when PrEP was not readily available. Some of them may be open about their PrEP status and have it disclosed on their profile. Information about PrEP and its implementation may also be more abundant in some neighboring countries such as Australia and Thailand [21] and that the latter had actually been enabling foreigners to have access to PrEP while traveling. Previous studies had confirmed that the internet was the main source of PrEP information [22]. Our results also highlighted the distinct pattern of information flow through different Web-based channels. Forum visitors had a lower awareness (33%), whereas mobile app users had a higher (68%) awareness level.

Conversely, PrEP awareness of MSM using online forums was particularly low. We noted that those who engaged in high-risk sexual activities, especially chemsex, had higher odds of knowing about PrEP. Chemsex was associated with a higher risk of HIV infection [23]. MSM who engaged in chemsex generally did not expect their partners to use condoms consistently. Such behaviors, together with other risk behaviors such as group sex, might have increased one’s perceived risk of HIV infection, thus contributing to their exploration of alternative means of HIV prevention. In other words, MSM with a low risk perception may think that their prevention measures were already sufficient [24].

Willingness to use PrEP, in general, was associated with an inclination to self-protect from HIV infection. They were not only more aware of their HIV status but also concerned about partner’s health status and behaviors, such as HIV status, STI history, and condom use and HIV testing habits. However, high-risk behavior per se was not associated with PrEP acceptance. This implies that MSM who were interested in PrEP were likely those who were concerned about their own health but not necessarily those with a higher actual risk [25]. Those who only had sexual relationships with their romantic partners may have a low perceived risk [26] that they did not think they would need PrEP. An unstable lifestyle pattern may imply an unstable income source that may become a barrier for them to access health care services, including PrEP. Other than cost, types of service providers may play a role in facilitating PrEP provision and affecting MSM’s acceptance toward PrEP, especially to those who work shifts. A separate study noted a lower acceptance (45%) if it was given free by local public health services [27]. Further investigation into MSM’s preferences on service provider is warranted.

As awareness and acceptance were not directly associated, an understanding of the pathways that link them is crucial. In our study, 2 specific forms of activities on these pathways, that is, chemsex and HIV testing, were found to be particularly important. MSM engaging in chemsex were paying little attention to their partner’s health status or condom use. As they are prone to HIV infection, efforts to scale up PrEP uptake among chemsex-practicing MSM are more likely to be successful in view of their higher awareness. The second important activity was HIV testing. Those who had undergone testing would be expecting the same from their partners. They were relatively more concerned about their own health and were more open to PrEP as an effective way for prevention. It has been shown that voluntary counseling and testing (VCT) clients who were aware of PrEP were more willing to start using it [11]. Such services could be a point of access to not only promote but also provide preparatory screening for renal function and hepatitis B infection in addition to HIV for efficient linkage to PrEP [12].

### Limitations

There were several limitations to this study such that extrapolation of its results is cautioned. First, some networking and behavioral questions referred to what happened in the preceding 3 months, so recall bias may exist. Second, sensitive questions such as sexual behaviors and drug use may induce social desirability bias. As our questionnaire was self-administered on the Web, the bias should have been minimized. On the contrary, the administration of the survey on the Web may cause self-selection bias, as only MSM inclined to respond to internet-based questions would participate in the study. Third, the main focus of the study was HIV testing behaviors, whereas PrEP was included as a new form of preventive intervention for which the awareness was assessed by a relatively simple trichotomized question. Its interpretation for inferring awareness about PrEP may not be robust for drawing a definitive conclusion. Finally, as PrEP was not available at that time through public services or implementation studies, willingness to use PrEP expressed by the respondents remained speculative.

### Conclusion

In conclusion, the networking channels were essential in affecting MSM’s awareness of PrEP in the community. Their low awareness warrants the specific development of community education, not only for information provision but also for the linkage to access channels, should it become available. In the future, when PrEP studies are piloted, collaboration with VCT services would be needed, and subjects’ sexual health needs, especially STI induced by chemsex and condomless sex, should be well addressed. Although VCT services could be convenient starting points, outreach activities targeting MSM engaging in chemsex would be good opportunities for enrolling risk-taking individuals to use PrEP. Such high-risk networks could indeed be an effective platform for developing HIV preventive interventions. The linkage between awareness and acceptance of PrEP highlighted the public health importance of taking on a targeted approach in identifying high-risk individuals for accessing PrEP.

## Appendix

Multimedia Appendix 1 Brief description of pre-exposure prophylaxis used in the study.

## Abbreviations

aOR: adjusted odds ratio
LBSN: location-based social network
MSM: men who have sex with men
OR: odds ratio
PrEP: pre-exposure prophylaxis
STI: sexually transmitted infection
VCT: voluntary counseling and testing
