# Supplementary material for: Bridging Awareness and Acceptance of Pre-Exposure Prophylaxis Among Men Who Have Sex With Men and the Need for Targeting Chemsex and HIV Testing: Cross-Sectional Survey
Source: JMIR Public Health Surveill. 2019 Jul 3;5(3):e13083. doi: 10.2196/13083 (PMC6636239; doi:10.2196/13083)
Supplement: Multimedia Appendix 1 [file publichealth_v5i3e13083_app1.pdf]

Supplementary file 1 Brief description of PrEP

Original text

接觸前預防性投藥(PrEP, Pre-Exposure Prophylaxis)是一種以服用藥物作預防愛滋病感染的方法。

外國研究顯示，使用投藥能提供四成至九成的額外保護作用。

在接受投藥前，用者須進行數項血液測試以確定身體狀況是適合接受投藥。

在開始接受投藥後，需要每一至三個月覆診一次，而部份血液測試需於每次覆診時重複進行。

使用接觸前預防性投藥，亦必須在性行為時使用安全套。

Translated version

PrEP is a means to prevent HIV infection by taking medicines.

Results from overseas studies showed that PrEP could offer 40-90% extra protection.

Before taking PrEP, users should undergo multiple blood tests to confirm one's suitability of using PrEP.

While on PrEP, user should seek medical advice for every 1-3 months. Some blood tests should be conducted at every clinic visit.

Condoms use is advised for sexual activities even on PrEP.
